# Predicting Breast Cancer in Chinese Women Using Machine Learning Techniques: Algorithm Development

**DOI:** 10.2196/17364

**Published:** 2020-06-08

**Authors:** Can Hou, Xiaorong Zhong, Ping He, Bin Xu, Sha Diao, Fang Yi, Hong Zheng, Jiayuan Li

**Affiliations:** 1 Department of Epidemiology and Biostatistics West China School of Public Health and West China Fourth Hospital Sichuan University Chengdu China; 2 Department of Head, Neck and Mammary Gland Oncology, Cancer Center West China Hospital Sichuan University Chengdu China; 3 Laboratory of Molecular Diagnosis of Cancer, Clinical Research Center for Breast West China Hospital Sichuan University Chengdu China

**Keywords:** machine learning, XGBoost, random forest, deep neural network, breast cancer

## Abstract

**Background:**

Risk-based breast cancer screening is a cost-effective intervention for controlling breast cancer in China, but the successful implementation of such intervention requires an accurate breast cancer prediction model for Chinese women.

**Objective:**

This study aimed to evaluate and compare the performance of four machine learning algorithms on predicting breast cancer among Chinese women using 10 breast cancer risk factors.

**Methods:**

A dataset consisting of 7127 breast cancer cases and 7127 matched healthy controls was used for model training and testing. We used repeated 5-fold cross-validation and calculated AUC, sensitivity, specificity, and accuracy as the measures of the model performance.

**Results:**

The three novel machine-learning algorithms (XGBoost, Random Forest and Deep Neural Network) all achieved significantly higher area under the receiver operating characteristic curves (AUCs), sensitivity, and accuracy than logistic regression. Among the three novel machine learning algorithms, XGBoost (AUC 0.742) outperformed deep neural network (AUC 0.728) and random forest (AUC 0.728). Main residence, number of live births, menopause status, age, and age at first birth were considered as top-ranked variables in the three novel machine learning algorithms.

**Conclusions:**

The novel machine learning algorithms, especially XGBoost, can be used to develop breast cancer prediction models to help identify women at high risk for breast cancer in developing countries.

## Introduction

In China, female breast cancer is the most prevalent malignant tumor affecting women, and its incidence is still increasing. According to the National Central Cancer Registry of China, more than 279,000 women were diagnosed with breast cancer in 2014, with a corresponding age-adjusted incidence rate of 28.77 per 100,000 [1]. The large number of breast cancer cases in China has resulted in a tremendous disease burden. In 2016, there were over 2 million disability-adjusted life years (DALYs) and 70,000 deaths due to breast cancer in China, accounting for approximately 15% of global DALYs and 13% of global deaths due to breast cancer [2]. Therefore, breast cancer is a major public health issue in China.

Breast cancer screening has proven to be an effective approach for breast cancer control. Several randomized controlled trials have shown that breast cancer screening can help detect breast cancer at an early stage and improve disease outcomes [3,4]. In many developed countries, population-based breast cancer screening programs have been implemented for several decades and brought positive results [5]. Nevertheless, due to the relatively low incidence rate, large population, and limited medical resources, population-based breast cancer screening is not feasible in China [6]. Therefore, some researchers have proposed risk-based breast cancer screening, considered to be cost-effective and more suitable for low- and middle-income countries like China [7].

Successful implementation of risk-based breast cancer screening largely relies on a breast cancer prediction model to accurately identify high-risk people before screening, but there is currently no suitable breast cancer prediction model for Chinese women. Some well known and commonly used breast cancer prediction models like the Gail and Tyrer-Cuzick models were developed based on women living in western countries, and their performance in Chinese women is unsatisfactory [8]. Hence, there is an urgent need to develop a breast cancer prediction model specifically for Chinese women.

Despite conventional statistical methods and some traditional machine learning algorithms (eg, logistic regression [LR]), modern machine learning has become an alternative approach for developing prediction models. Different from traditional prediction models where relationships between dependent and independent variables are predefined using prior knowledge, modern machine learning can automatically learn the underlying patterns of the data without any implicit assumptions [9]. This is especially the case for tree-based machine learning algorithms such as decision trees. These algorithms only make weak assumptions about the form of the mapping function and are therefore free to learn any functions underlying the training data and can deal with nonlinear relationships and higher order interactions between variables [10], both of which are common challenges in the health care field. In contrast, as a form of parametric machine learning algorithm, an artificial neural network (ANN) also makes strong assumptions about the functional form but it can still be used for modeling nonlinear relationships and high-order interactions. This is mainly due to the use of nonlinear activation functions in ANN and the sufficient complexity (depth and number of neurons) of the networks [11].

The objectives of this study are to evaluate and compare the performance of four different machine learning algorithms on predicting breast cancer among Chinese women and choose the best machine learning algorithm to develop a breast cancer prediction model. We used three novel machine learning algorithms in this study: extreme gradient boosting (XGBoost), random forest (RF), and deep neural network (DNN), with traditional LR as a baseline comparison.

## Methods

### Dataset and Study Population

In this study, we used a balanced dataset for training and testing the four machine learning algorithms. The dataset comprises 7127 breast cancer cases and 7127 matched healthy controls. Breast cancer cases were derived from the Breast Cancer Information Management System (BCIMS) at the West China Hospital of Sichuan University. The BCIMS contains 14,938 breast cancer patient records dating back to 1989 and includes information like patient characteristics, medical history, and breast cancer diagnosis [12]. West China Hospital of Sichuan University is a government-owned hospital and has the highest reputation in terms of cancer treatment in Sichuan province; the cases derived from the BCIMS are representative of breast cancer cases in Sichuan [12].

Han Chinese women living in Sichuan province who were first diagnosed with primary breast cancer between 2000 and 2017 were included (12,175 cases were included and 2763 cases were excluded). We excluded cases of patients with mental disorder and aged younger than 30 years or older than 70 years (11,916 cases were included and 259 cases were excluded). For the remaining cases, those containing missing values (4,771 cases) or contradictory data (18 cases; eg, age at first birth < age of menarche) were also excluded. Finally, a total of 7127 cases were eligible and included in the study. For each eligible breast cancer case, a main residence (urban or rural area) matched healthy control was selected from women who participated in the Breast Cancer Screening Cohort in Sichuan from 2009 to 2017. The screening project was launched at Chengdu Women’s and Children’s Central Hospital and Shuangliu Maternal and Child Health Hospital with the purpose of providing free screening for breast cancer, cervical cancer, and reproductive tract infections to women aged between 30 and 70 years.

A total of 13,607 women living in Chengdu and 15,704 women living in Shuangliu county were recruited in the cohort, representing Han Chinese women living in the developed and less developed regions of Sichuan province, respectively. Eligibility criteria for controls were Han Chinese, living in Sichuan province, confirmed to have no breast cancer, and without mental disorder and other malignant tumors. If a woman had more than one screening record, the most recent record was used.

### Variable Selection

For the controls, a standard questionnaire was used to collect demographic and risk factor information, whereas for the cases, the corresponding data were directly extracted from the BCIMS. Independent variables that were included in the machine learning models were selected based on the following criteria: (1) they must be potential or known breast cancer risk factors and (2) they must be collected using the same measurement methods and have the same definitions in the cases and controls. A total of 10 variables, including 3 demographic factors, 6 reproductive history factors, and family history of breast cancer, met the above criteria and were selected for classification. Table 1 shows the details of the 10 independent variables selected along with the outcome variable.

**Table 1:** Descriptions and details of the variables included in the machine learning algorithms.

Variable	Types of variable	Description
Main residence	Categorical variable^a^	Urban area (0), rural area (1)
Menopausal status	Categorical variable	Premenopause (0), postmenopause (1)
Age in years	Discrete variable	Age at breast cancer diagnosis or screening
BMI (kg/m^2^)	Continuous variable	BMI at breast cancer diagnosis or screening
Age of menarche	Discrete variable	Age at first menstruation
Duration of reproductive life span	Discrete variable	Premenopausal women: current age – age of menarche; postmenopausal women: menopause age – age of menarche
Pregnancy history	Categorical variable	No (0), yes (1)
Number of live births	Discrete variable	Live births is defined as births of children who showed any sign of life
Age at first birth	Discrete variable	Age of women at birth of first child (for women with no live birth, this value equals 99)
Family history of breast cancer	Categorical variable	First-degree or second-degree female relatives had breast cancer: no (0), yes (1)
Case-control status (outcome variable)	Categorical variable	Control (0), case (1)

^a^Categorical variables were converted into one-hot encoding before being provided to machine learning algorithms.

### Machine Learning Algorithms

In this study, three novel machine learning algorithms (XGBoost, RF, and DNN) along with a baseline comparison (LR) were evaluated and compared.

XGBoost and RF both belongs to ensemble learning, which can be used for solving classification and regression problems. Different from ordinary machine learning approaches where only one learner is trained using a single learning algorithm, ensemble learning consists of many base learners. The predictive performance of a single base learner is merely slightly better than random guess, but ensemble learning can boost them to strong learners with high prediction accuracy by combination [13]. There are two methods to combine base learners: bagging and boosting. The former is the base of RF while the latter is the base of XGBoost. In RF, decision trees are used as base learners and bootstrap aggregating, or bagging, is used to combine them [14]. XGBoost is based on the gradient boosted decision tree (GBDT), which uses decision trees as base learners and gradient boosting as combination method. Compared with GBDT, XGBoost is more efficient and has better prediction accuracy due to its optimization in tree construction and tree searching [15].

DNN is an ANN with many hidden layers [16]. A standard ANN is made up of an input layer, several hidden layers, and an output layer, and each layer contains multiple neurons. Neurons in the input layer receive values from the input data, neurons in other layers receive weighted values from the previous layers and apply nonlinearity to the aggregation of the values [16]. The learning process is to optimize the weights using a backpropagation method to minimize the differences between predicted outcomes and true outcomes. In contrast to shallow ANN, DNN can learn more complex nonlinear relationships and is intrinsically more powerful [17].

### Hyperparameters Tuning, Model Development, and Algorithm Comparison

**Figure 1:**
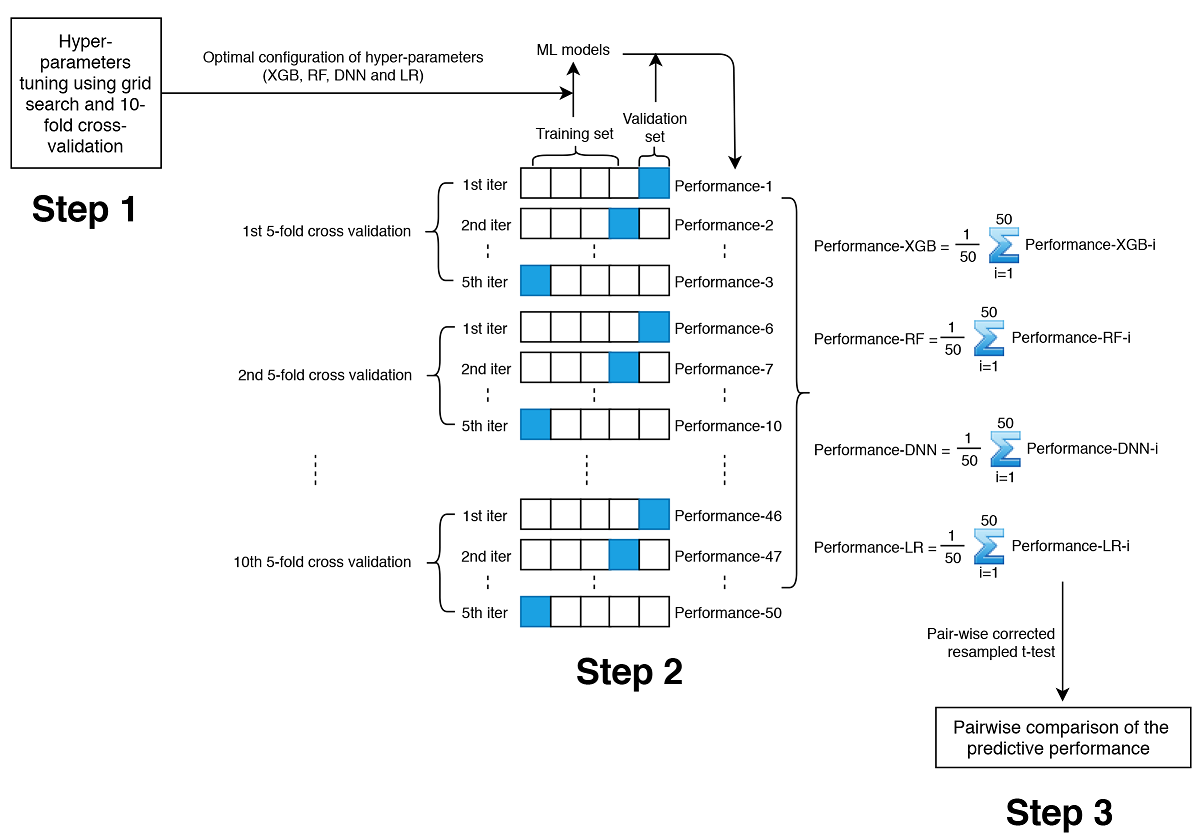
Process of model development and algorithm comparison. Step 1: hyperparameters tuning; step 2: model development and assessment; step 3: algorithm comparison. Performance metrics include area under the receiver operating characteristic curve, sensitivity, specificity, and accuracy.

A general overview of the model development and algorithm comparison process is illustrated in Figure 1. The first step was hyperparameters tuning, with the purpose of choosing the most optimal configuration of hyperparameters for each machine learning algorithm. In DNN and XGBoost, we introduced dropout and regularization techniques, respectively, to avoid overfitting, whereas in RF, we tried to reduce overfitting by tuning the hyperparameter min_samples_leaf. We conducted a grid search and 10-fold cross-validation on the whole dataset for hyperparameters tuning. The results of the hyperparameters tuning along with the optimal configuration of hyperparameters for each machine learning algorithm is shown in Multimedia Appendix 1.

Based on the optimal configuration of hyperparameters, the next step was model development and assessment. In this step, we used repeated 5-fold cross-validation. This method can avoid overfitting and increase robustness of the results. In each 5-fold cross-validation, the dataset was randomly divided into 5 folds with approximately equal sample size, where 4 folds were chosen as training set to develop the machine learning models while the remaining 1 fold was used as the validation set to calculate the model performance metrics (including area under the receiver operating characteristic curve [AUC], sensitivity, specificity, and accuracy). After 5 iterations, each fold (as well as each subject) was used as validation set exactly once. We repeated the whole 5-fold cross-validation process 10 times, and in each repetition, the division of the dataset was different.

The final step was algorithm comparison. For each machine learning algorithm, we summarized their predictive performance metrics generated from the second step using means and standard deviations and conducted pair-wise comparison using statistical tests. AUC was chosen as the primary measure of the predictive performance in our study.

RF and LR algorithms were implemented in Python 3.6 (Python Software Foundation) using scikit-learn (version 0.20.0). XGBoost and DNN algorithms were implemented in Python 3.6 using xgboost (version 0.80) and TensorFlow (version 1.10.0), respectively. Source code is shown in Multimedia Appendix 2.

### Variable Ranking

To have a deeper understanding of the three novel machine learning algorithms, we ranked all the variables based on their impact on AUCs. To do so, we repeated step 1 and 2 illustrated above, but in each iteration of the repeated 5-fold cross-validation, we successively permuted the values of the 10 variables in the testing set and calculated the corresponding decrease in the AUC (in percentage). Then the results were summarized and used for ranking each variable in the four machine learning algorithms. Detailed process is illustrated in Multimedia Appendix 3.

### Statistical Analysis

Characteristics of the cases and controls were described using medians (IQRs) for continuous or discrete variables and number (%) for categorical variables. For the comparison of characteristics between cases and controls, we used a Mann-Whitney *U* test for continuous or discrete variables and Pearson chi-square test for categorical variables, with a significance level of .05. As for the pair-wise comparison of the predictive performance of the machine learning algorithms, we used the pair-wise corrected resampled *t* test [18]. To counteract the issue of multiple comparisons, the significance level was adjusted to .008 using Bonferroni correction. All statistical analyses were conducted using SciPy (version 1.1.0), pandas (version 0.23.0), and NumPy (version 1.14.3) in Python 3.6.

## Results

As shown in Table 2, a total of 7127 breast cancer cases along with 7127 matched healthy controls were included in the dataset. Among the cases, 61.27% (4367/7127) were premenopausal women and 38.73% (2760/7127) were postmenopausal women, while among the controls, 63.80% (4547/7127) were premenopausal women and 36.20% (2580/7127) were postmenopausal women. Except for BMI, menarche age, main residence, and family history of breast cancer, all other features were significantly different between the cases and controls.

**Figure 2:**
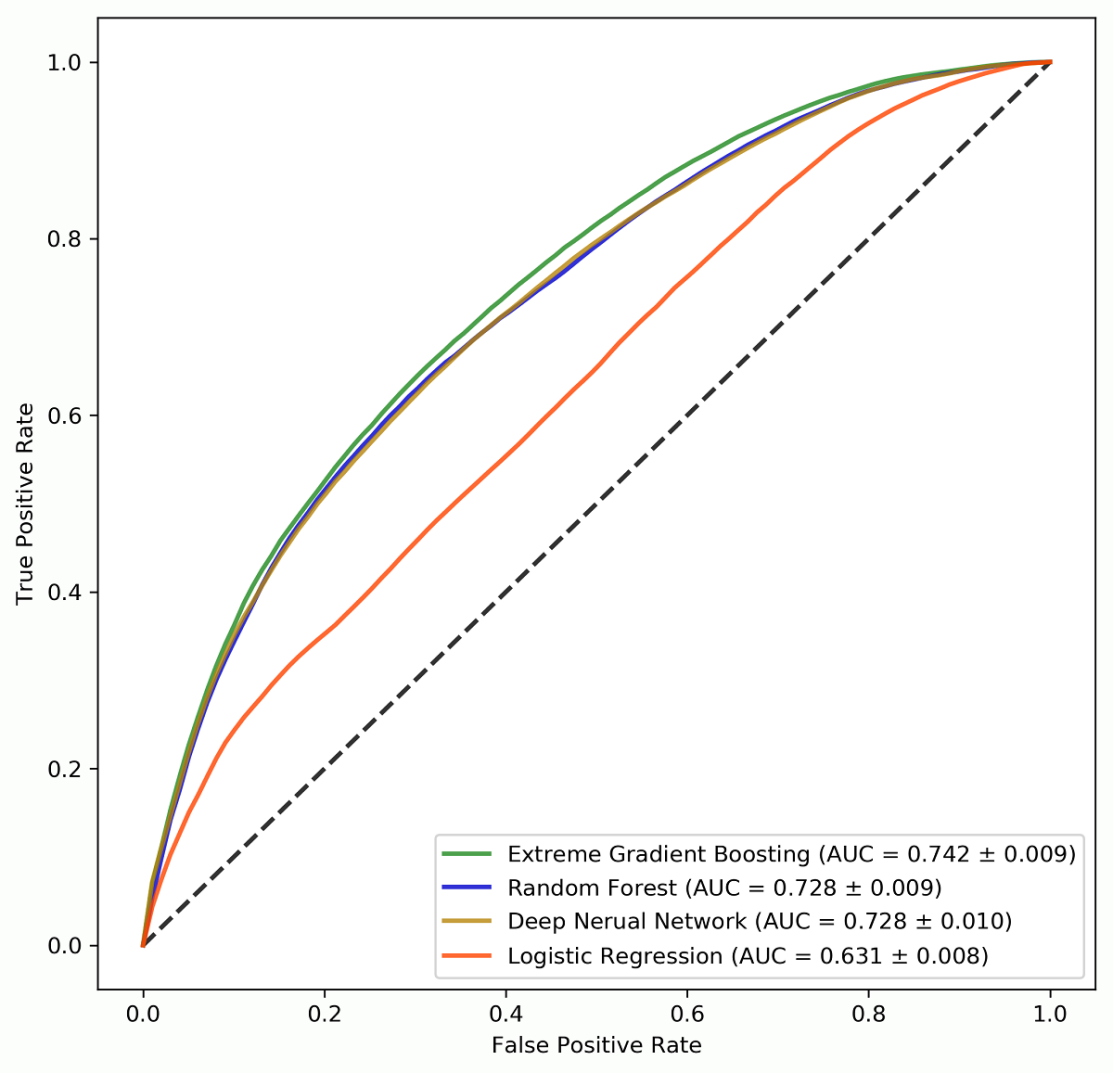
Mean receiver operating characteristic curves for the four machine learning algorithms.

The predictive performance of the four machine learning algorithms is shown in Figure 2 and Table 3, and the results of the pair-wise corrected resampled *t* test are shown in Multimedia Appendix 4. The three novel machine learning algorithms all achieved significantly higher AUCs than the linear LR algorithm (*P*<.001). To be more specific, XGBoost had a mean score of 0.742 in terms of AUC, followed by DNN (0.728), RF (0.728), and LR (0.631). XGBoost had significantly higher AUC than both DNN and RF (*P*<.001), whereas no statistically significant difference was found between the mean AUCs of DNN and RF (*P*=.98). Similarly, the mean sensitivity of LR (49.6%) was significantly lower compared with that of XGBoost (65.6%), DNN (64.2%), or RF (65.0%; *P*<.001). However, for XGBoost, DNN, and RF, their mean sensitivities were not significantly different from each other.

As for specificity, the four machine learning algorithms achieved similar results (LR: 66.1%; DNN: 67.9%; XGBoost: 68.6%; RF: 67.7%) and no statistically significant differences were found between them. Since we used a balanced dataset to train the models, we have also reported accuracy. Compared with XGBoost (67.1%), DNN (66.1%), and RF (66.3%), LR had the lowest accuracy (57.8%). Although XGBoost had the highest mean accuracy, there was no significant difference between the mean accuracy of XGBoost and RF (*P*=.34). The difference between the mean accuracy of DNN and RF was also statistically insignificant (*P*=.01).

**Figure 3:**
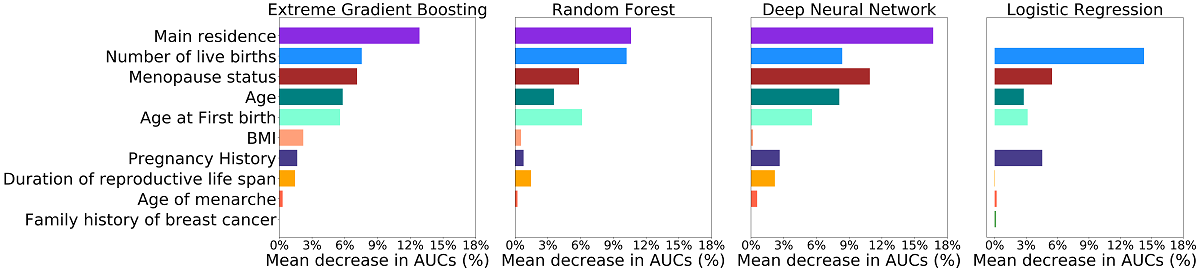
Variable rankings according to the mean area under the receiver operating characteristic curve decrease in percentage in the four machine learning algorithms.

Figure 3 presents the variable rankings according to the mean decrease in AUCs in different machine learning algorithms. XGBoost, RF, and DNN were very similar in variable rankings, although some discrepancies did exist. In all the three novel machine learning algorithms, main residence, number of live births, menopause status, age, and age at first birth were considered as top-ranked variables. Since the cases and controls were matched by main residence, linear LR prioritized all other variables over main residence. Moreover, pregnancy history, which was not present in top-ranked variables for the three novel machine learning algorithms, was prioritized over age and age at first birth in LR.

**Table 2:** Characteristics of case and control participants.

Variable		Control	Case	*P* value^a^
Age in years, median (IQR)		47 (41-53)	47 (42-54)	<.001
BMI (kg/m^2^), median (IQR)		22.83 (20.96-24.77)	22.89 (20.94-24.97)	.16
Age of menarche, median (IQR)		14 (13-15)	14 (13-15)	.23
Duration of reproductive lifespan, median (IQR)		31 (27-34)	32 (27-35)	<.001
Number of live births, median (IQR)		1 (1-1)	1 (1-2)	<.001
Age at first birth^b^, median (IQR)		24 (22-26)	24 (23-26)	<.001
**Main residence, n (%)**				>.99
	Urban area	3994 (56.04)	3994 (56.04)	
	Rural area	3133 (43.96)	3133 (43.96)	
**Pregnancy history, n (%)**				<.001
	No	201 (2.82)	14 (0.20)	
	Yes	6926 (97.18)	7113 (99.80)	
**Family history of breast cancer, n (%)**				.17
	No	7010 (98.36)	6988 (98.05)	
	Yes	117 (1.64)	139 (1.95)	
**Menopausal status, n (%)**				<.01
	Premenopause	4367 (61.27)	4547 (63.80)	
	Postmenopause	2760 (38.73)	2580 (36.20)	

^a^*P* values are derived from Mann-Whitney *U* test or Pearson chi-square test.

^b^Only women with at least one birth are summarized.

**Table 3:** Performance of the four machine learning algorithms on predicting breast cancer risk.

Algorithm	AUC^a^, mean (SD)	Sensitivity^b^, mean (SD)	Specificity^b^, mean (SD)	Accuracy^b^, mean (SD)
Extreme gradient boosting	0.742 (0.009)	0.656 (0.017)	0.686 (0.012)	0.671 (0.009)
Random forest	0.728 (0.009)	0.650 (0.016)	0.677 (0.015)	0.663 (0.010)
Deep neural network	0.728 (0.010)	0.642 (0.037)	0.679 (0.033)	0.661 (0.010)
Logistic regression	0.631 (0.008)	0.496 (0.020)	0.661 (0.021)	0.578 (0.008)

^a^AUC: area under the receiver operating characteristic curve.

^b^Sensitivity, specificity, and accuracy were calculated using the default cutoff value (0.5).

## Discussion

### Principal Findings

In this study, we used four machine learning algorithms to develop breast cancer prediction models to identify Chinese women at high risk of breast cancer, based on 10 breast cancer risk factors. Their predictive performances were evaluated and compared, and the results indicated that compared with traditional LR, all three novel machine learning algorithms achieved better performance and improved the AUC by 0.11 at most. Among the three novel machine learning algorithms, XGBoost outperformed RF and DNN, with mean AUC and accuracy of 0.74 and 67.1%, respectively.

Among the three novel machine learning algorithms used in this study, XGBoost is the most up to date. Recently, XGBoost has dominated many data mining competitions for structured datasets and gained much attention in the machine learning field. Some previous studies have shown that XGBoost has better performance on low-dimensional data than high-dimensional data [19,20]. RF, on the other hand, is more suitable for high-dimensional data due to its implicit feature selection characteristic [21]. As for DNN, it is more commonly used for prediction with unstructured data and data with complex structure [22]. Therefore, the results of this study were expected, since the structure of our dataset agrees with the XGBoost algorithm most.

Meanwhile, XGBoost was also faster than DNN and RF. The average times of training XGBoost, DNN, and RF using the current dataset were 0.20 seconds, 21.38 seconds, and 0.61 seconds, respectively (CPU: Intel i7-4790; GPU: GeForce GTX 970). Given the above, XGBoost is no doubt the optimal choice for developing a breast cancer prediction model using traditional breast cancer risk factors. Nevertheless, DNN and RF are also powerful machine learning algorithms and could be considered for developing a breast cancer prediction model in other circumstances. For example, if the dataset contains high-dimensional genetic data, RF is very likely to be the best choice. As for DNN, it can be used to predict breast cancer based on breast ultrasound or mammogram images when integrated with a convolutional neural network (CNN).

Although the predictive accuracy of the novel machine learning algorithms is still imperfect, some remarkable improvements have been made compared with previous breast cancer risk prediction models. The Gail model is the most well-known breast cancer risk assessment tool. It uses six breast cancer risk factors to estimate a women’s risk of developing breast cancer, including patient demographics, reproductive history, personal medical history, and family history of breast cancer [23]. Among these risk factors, four risk factors (age, age of menarche, age at first birth, and family history of breast cancer) are also present in this study. A recent meta-analysis of 26 studies reported a pooled AUC of 0.59 (95% CI 0.57 to 0.61) for the Gail model, which is significantly lower than the AUCs of XGBoost, RF, and DNN algorithms in our study. The authors also conducted subgroup analyses by geographic region, and the results revealed that the pooled AUC for the Gail model in Asian women was even lower (0.55, 95% CI 0.52 to 0.58).

Another famous breast cancer risk prediction model is the Rosner-Colditz model. This model is more complex than the Gail model and includes some key risk factors omitted from the Gail model such as type of menopause, BMI, and duration and type of postmenopausal hormone therapy used [24]. A validation study conducted by Rosner et al [25] using the dataset from the California Teachers Study revealed that the Rosner-Colditz model achieved an overall AUC of 0.59, higher than the AUC of the Gail model when applied in the same dataset. Different from the machine learning models in this study, classical breast cancer risk prediction models like the Gail and Rosner-Colditz models put more of an emphasis on estimating the probability of having breast cancer in a defined age interval instead of identifying breast cancer cases from noncases. In addition, all these models are based on an implicit assumption that each risk factor has a linear relationship with breast cancer and therefore largely ignore complex nonlinear relationships between risk factors and breast cancer and interactions between risk factors.

Many studies have been conducted to evaluate the performance of machine learning algorithms for breast cancer prediction. However, the majority of these studies used medical imaging data to develop the models, and only few focused on prediction with breast cancer risk factors. Shieh et al [26] conducted a nested case-control study in the United States to investigate the predictive performance of combining the Breast Cancer Surveillance Consortium (BCSC) risk model with an 83-single nucleotide polymorphism (SNP)–based polygenic risk score (PRS) in an LR model. They reported that compared with using the BCSC model alone, the LR model combining BCSC risk factors and PRS increased the AUC from 0.62 to 0.65. Dite et al [27] also evaluated the performance of an LR model that combined several breast cancer prediction models with a risk score based on 77 SNPs. They reported that the LR model using the absolute risk from the Breast and Ovarian Analysis of Disease Incidence and Carrier Estimation Algorithm model and the SNP-based score achieved the best performance (AUC: 0.70). Anothaisintawee et al [28] developed an LR model for predicting breast cancer risk among Thai women using the dataset from a cross-sectional study. The variables used in building the model included age, menopausal status, BMI, and use of oral contraceptives. The LR model achieved AUCs of 0.65 (95% CI 0.64 to 0.65) and 0.61 (95% CI 0.51 to 0.71) on the internal validation and external validation datasets, respectively. Zheng et al [29] also developed an LR model for predicting breast cancer. They derived the dataset from a case-control study in China and used 12 SNPs along with age at menarche, age at first live birth, waist-to-hip ratio, family history of breast cancer, and a previous diagnosis of benign breast disease for model building. The final LR model had an AUC of 0.63, which the authors believe is inadequate for cancer diagnosis and screening. Zhao et al [30] conducted the only study that evaluated the performance of a machine learning algorithm other than LR. They built an ANN model with one hidden layer for Chinese women using a cross-sectional dataset. In the test set, the ANN model achieved an AUC of 0.71 (95% CI 0.66 to 0.76). Compared with the previous machine learning models, our novel machine learning model achieved higher AUCs. More importantly, our model only requires 10 breast cancer risk factors, which can be easily collected in a cost-effective manner.

One of the major disadvantages of machine learning algorithms is that they are hard to interpret, especially for DNN. In our study, we tried to investigate the independent effects of each variable on breast cancer prediction. XGBoost, DNN, and RF all identified main residence as the most important variable in the models. This finding indicates significant interactions between main residence and other risk factors, but we cannot determine how main residence is interacting with other risk factors. A possible explanation for the interactions is the differences in lifestyle and environmental conditions in rural and urban area. A previous cross-sectional survey conducted in China also reported that there were some differences in the breast cancer risk factors between urban and rural populations [31]. Other top-ranked variables in the machine learning models are also considered to be important breast cancer risk factors in Chinese women [32,33].

It is also recognized that machine learning algorithms are vulnerable to overfitting. To address this issue, we used repeated k-fold cross-validation method to evaluate the performance of the models. Meanwhile, we also tried to reduce overfitting by choosing appropriate hyperparameters and using regularization techniques. Another limitation of using machine learning algorithms for breast cancer prediction is that absolute risk of having breast cancer cannot be estimated. However, it is not necessarily the case that machine learning models are less useful than traditional absolute breast cancer prediction models. In some circumstances (eg, risk-based breast cancer screening), the primary objective is to identify women with high risk from those with lower risk rather than informing personalized absolute risk. Therefore, in that case machine learning models with higher discriminatory accuracy would be more useful.

### Strengths and Limitations

To the best of our knowledge, this is the first study applying the novel machine learning algorithms to breast cancer prediction. The strengths of our study include using a large balanced dataset for model training and conducting repeated k-fold cross-validation for model evaluation. Nevertheless, our study still has several limitations. First, since an observational study design was adopted to derive the dataset, the influence of selection bias cannot be omitted. A better choice would be deriving the dataset from a large cohort study with sufficient breast cancer cases. However, considering the incidence of breast cancer, using such dataset would raise the issue of imbalanced classes that require statistical techniques to deal with. Second, due to the limitations of the dataset, only 10 breast cancer risk factors were chosen to build the model and some important risk factors like breastfeeding and history of other breast diseases were excluded, which may have influenced the performance of the models. In addition, our machine learning models were trained on cases and noncases from Sichuan province in southwest China and therefore may not be useful for women living in other parts of China. Furthermore, although we validated our models using the cross-validation method, external validation using an independent dataset was not performed in our study.

Currently, we have developed our final breast cancer prediction model using XGBoost and implemented it in a mobile phone app. Our next step is to validate this prediction model in a real-world setting and upgrade the model by including more risk factors and potentially medical imaging data. After external validation, we also plan to conduct a large observational study to evaluate the cost-effectiveness of applying this model in risk-based breast cancer screening among Chinese women.

### Conclusion

Our study has shown that all three novel machine learning algorithms achieved better discriminatory accuracy on identifying women at high risk of breast cancer than LR, and XGBoost is the best choice for developing a breast cancer prediction model using breast cancer risk factors. We have successfully developed and validated a breast cancer prediction model for Chinese women using XGBoost, but external validation is needed before implementation.

## Appendix

Multimedia Appendix 1 Hyperparameter tuning results and optimal configuration of hyperparameters for each machine learning algorithm.

Multimedia Appendix 2 Python code and outputs.

Multimedia Appendix 3 Detailed process of variable ranking.

Multimedia Appendix 4 Results of the pairwise comparison of the model predictive performance.

## Abbreviations

ANN: artificial neural network
AUC: area under the receiver operating characteristic curve
BCIMS: Breast Cancer Information Management System
BCSC: Breast Cancer Surveillance Consortium
DALY: disability-adjusted life year
DNN: deep neural network
GBDT: gradient boosted decision tree
LR: logistic regression
PRS: polygenic risk score
RF: random forest
SNP: single nucleotide polymorphism
XGBoost: extreme gradient boosting
