# Supplementary material for: Predicting Breast Cancer in Chinese Women Using Machine Learning Techniques: Algorithm Development
Source: JMIR Med Inform. 2020 Jun 8;8(6):e17364. doi: 10.2196/17364 (PMC7308891; doi:10.2196/17364)
Supplement: Multimedia Appendix 1 [file medinform_v8i6e17364_app1.pdf]

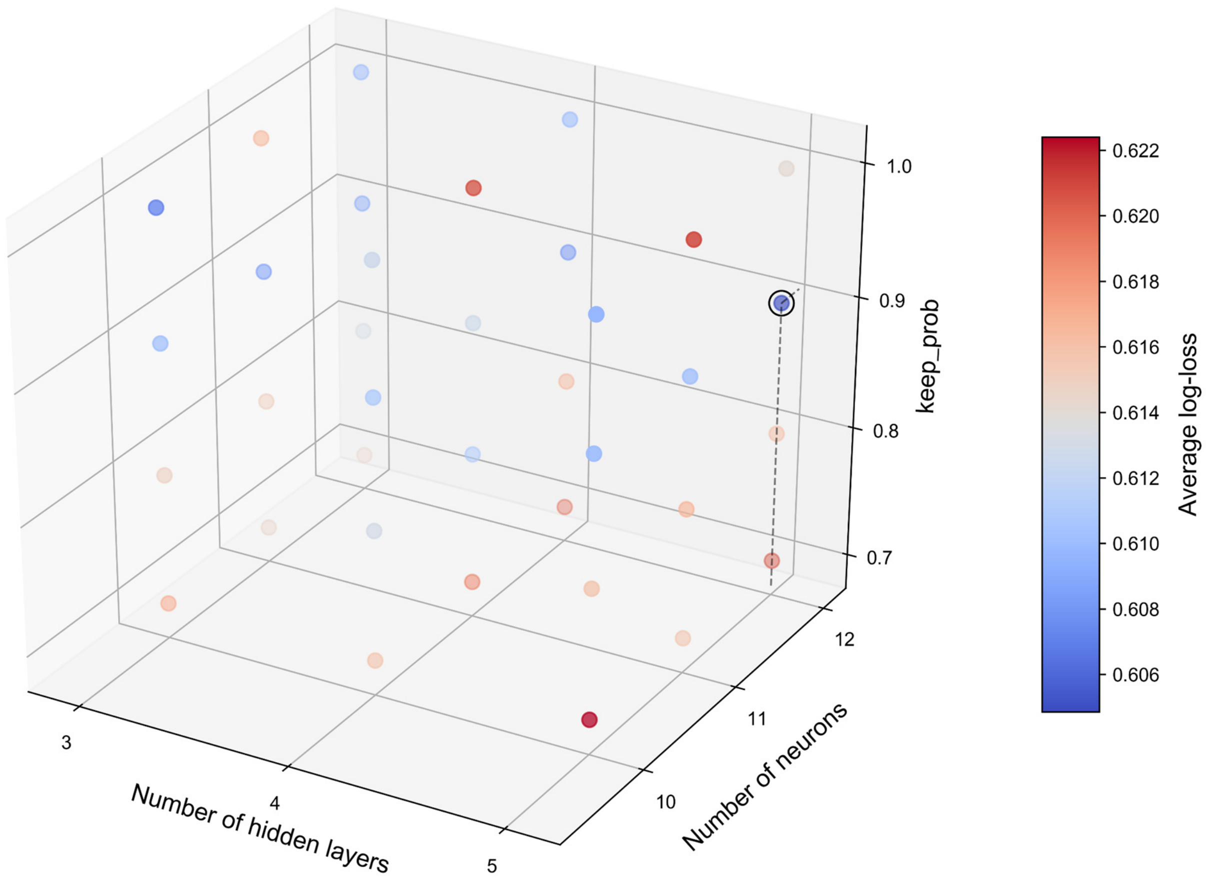

Figure: Results of the hyper-parameters tuning (Deep Neural Network)

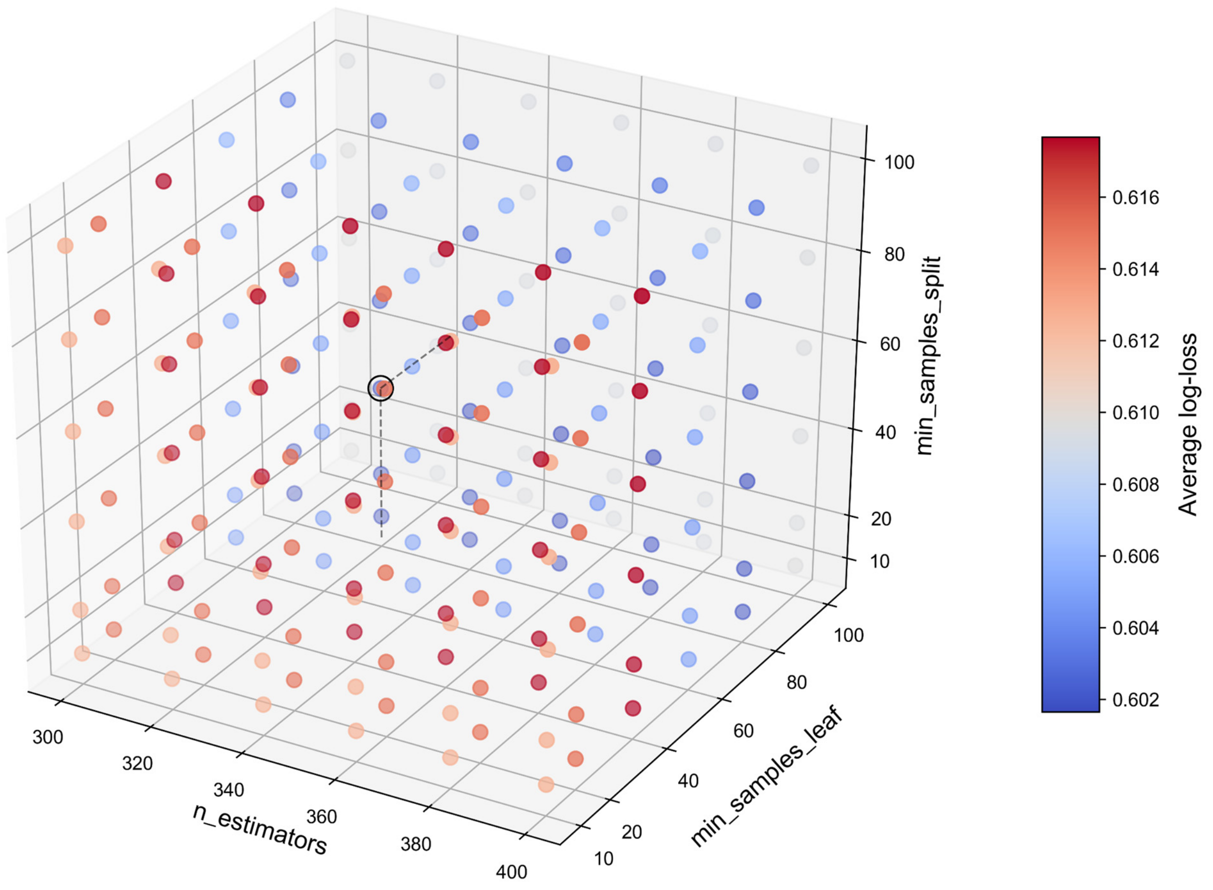

Figure: Results of the hyper-parameters tuning (Random Forests)

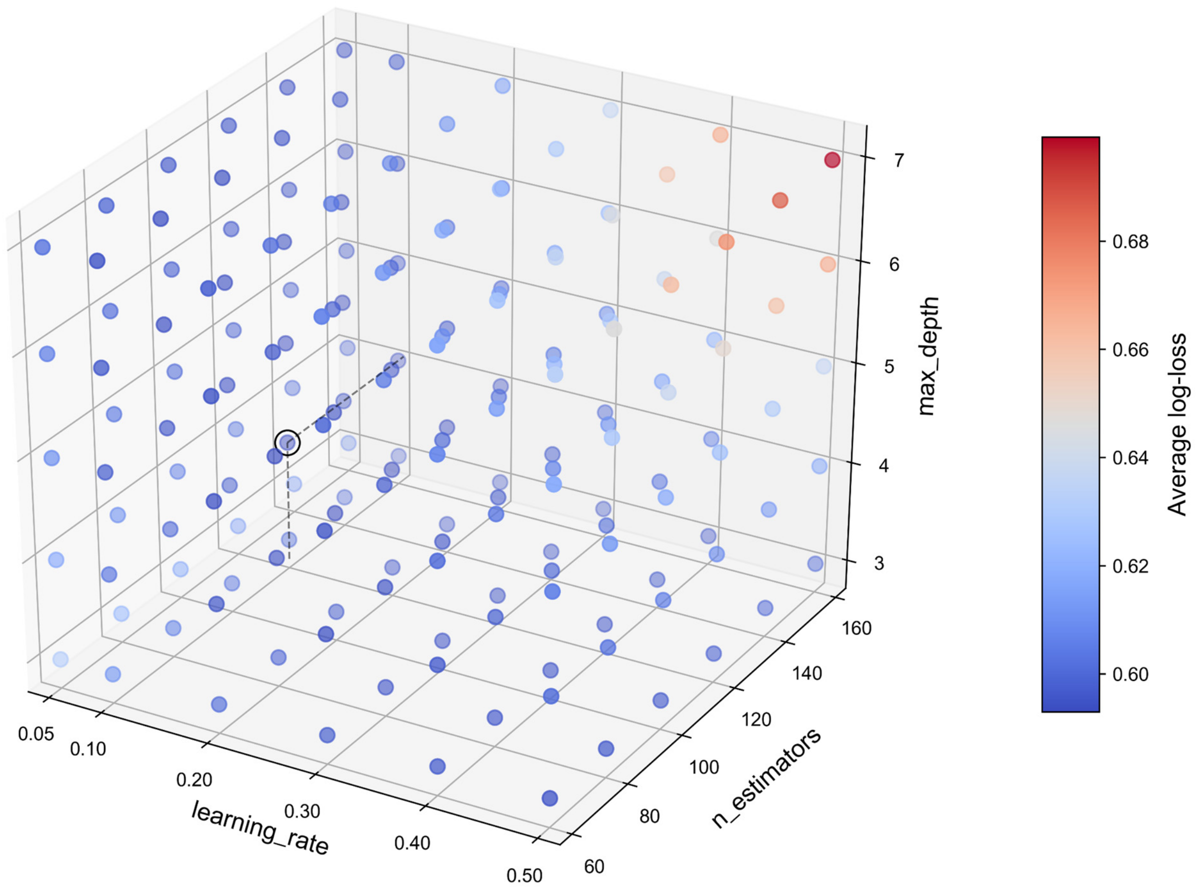

Figure: Results of the hyper-parameters tuning (Extreme Gradient Boosting-1)

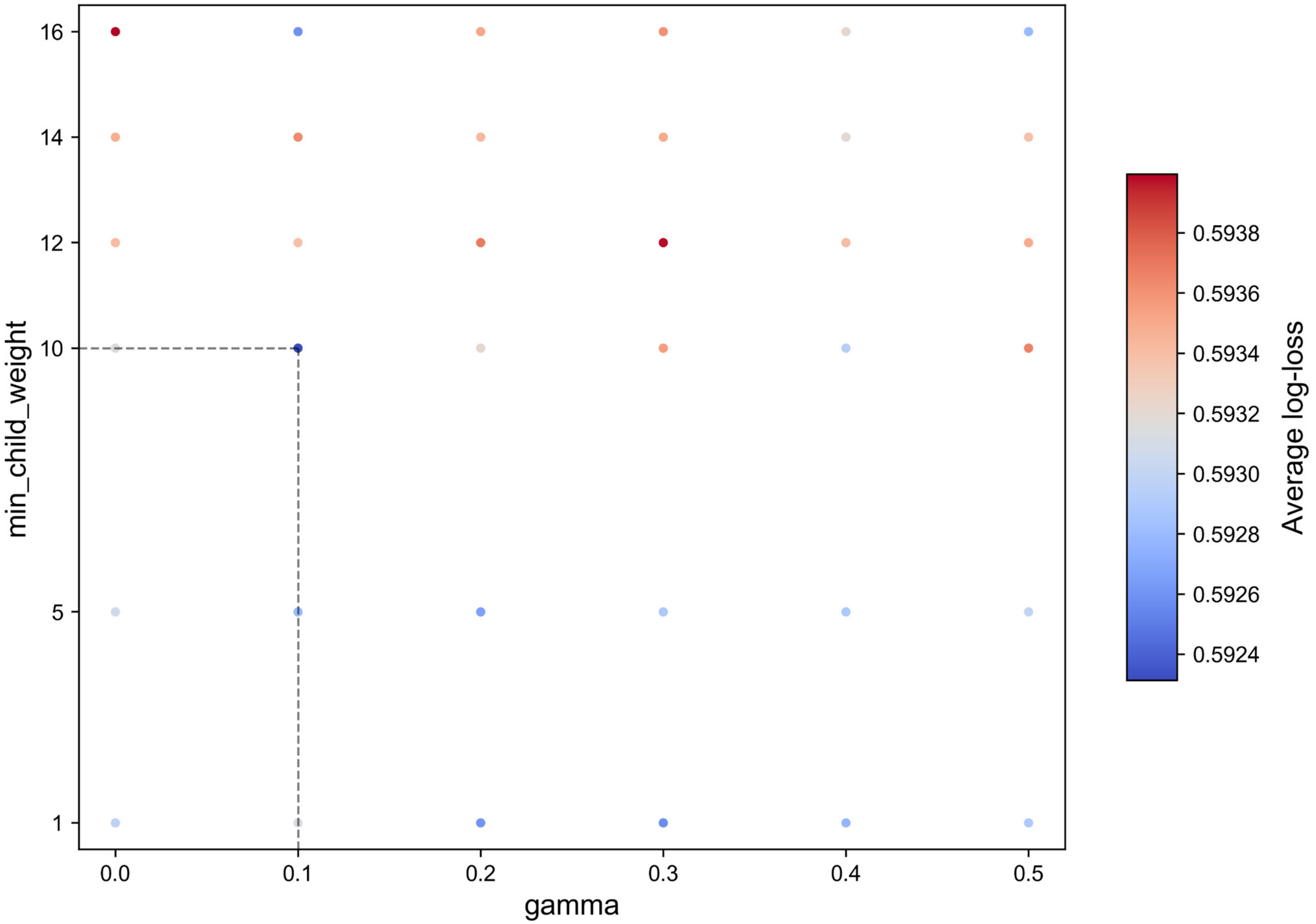

Figure: Results of the hyper-parameters tuning (Extreme Gradient Boosting-2)

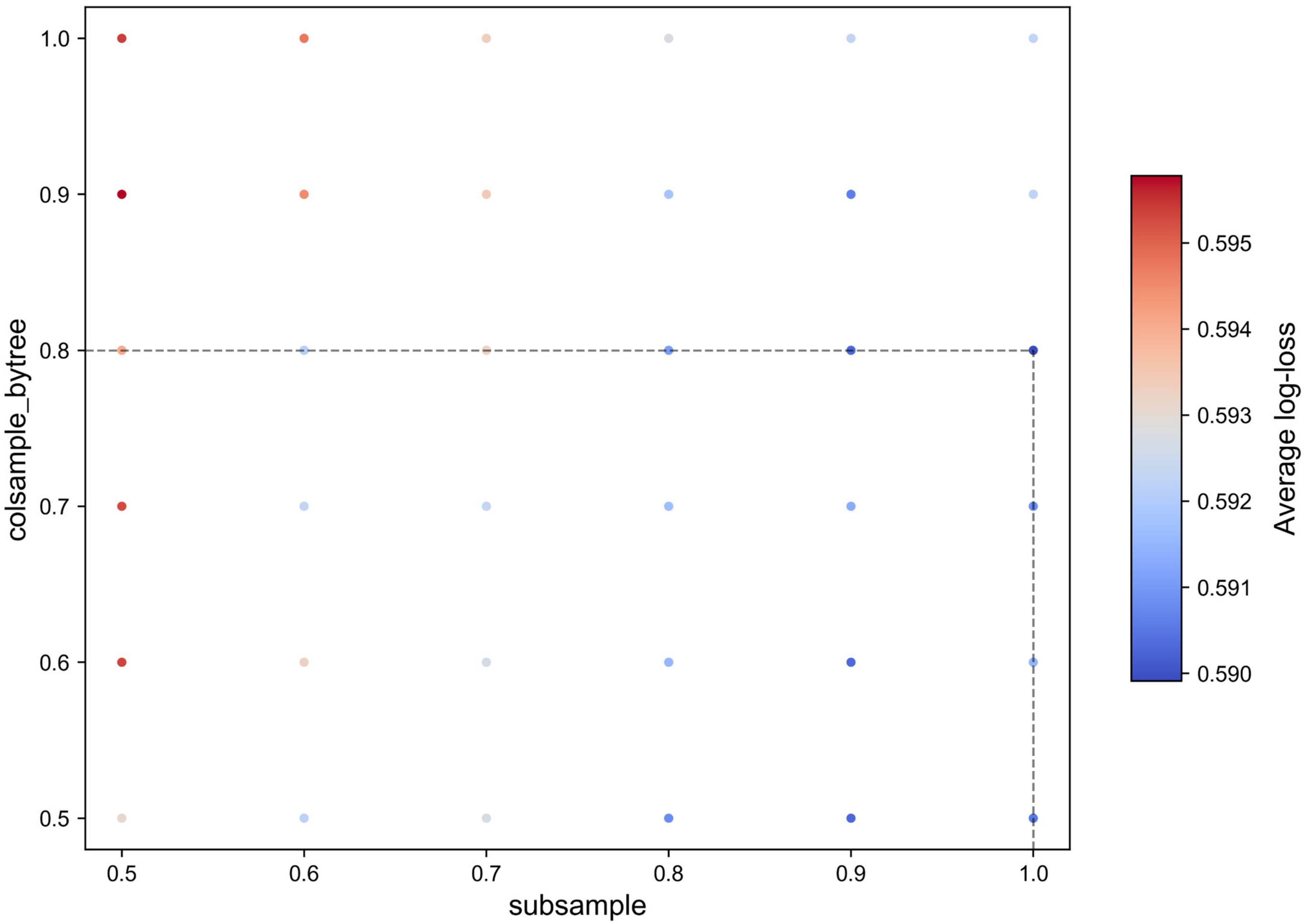

Figure: Results of the hyper-parameters tuning (Extreme Gradient Boosting-3)

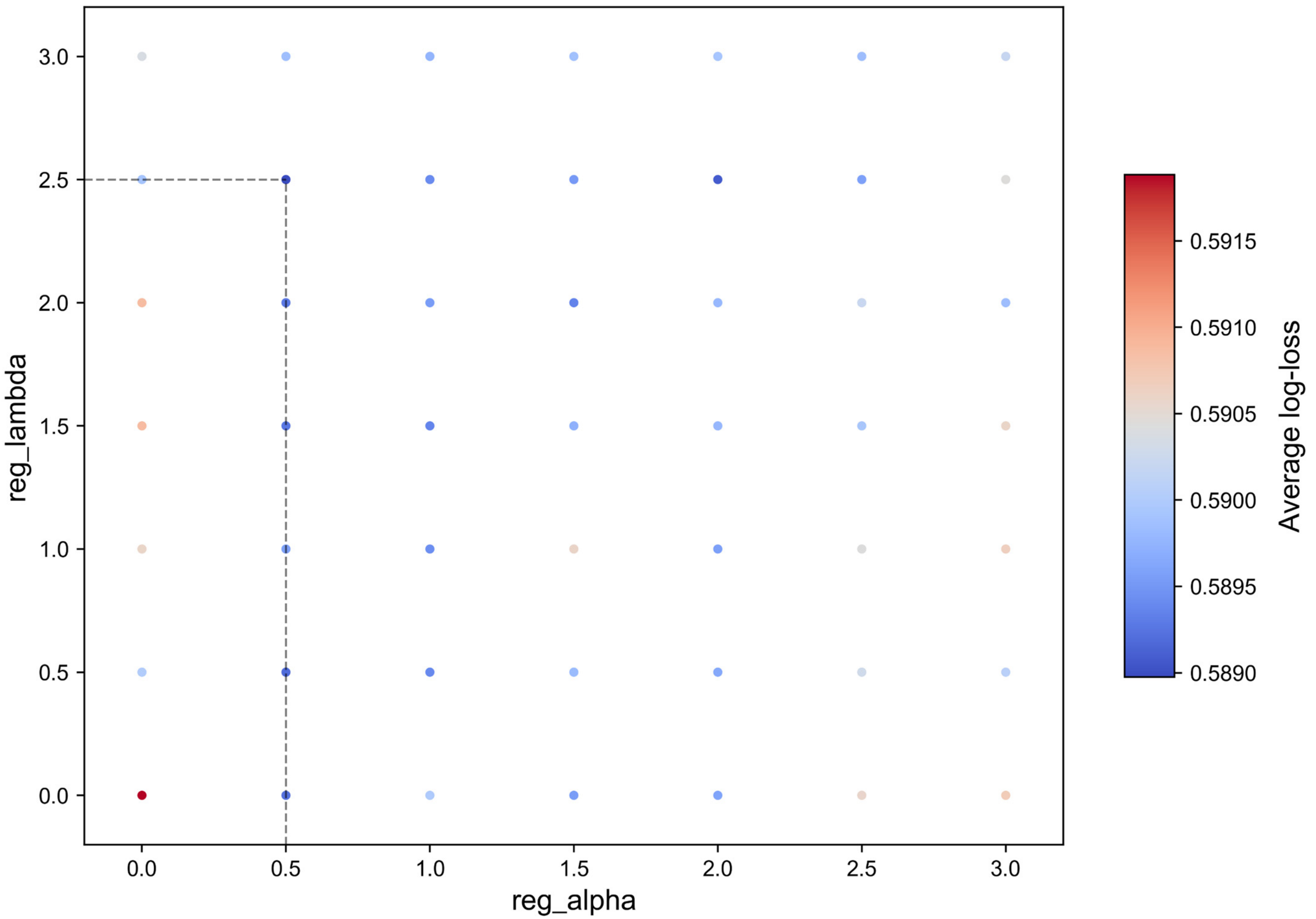

Figure: Results of the hyper-parameters tuning (Extreme Gradient Boosting-4)

Table: Configuration of hyper-parameters in each machine-learning algorithm (hyper-parameters that were tuned are highlighted in red)

Machine-learning algorithm	Hyper-parameter name	Description	Value
Random Forest†	criterion	Function to measure the quality of a split	Gini
	n_estimators	Number of trees in the forest	320
	max_depth	Maximum depth of the tree	None
	min_samples_split	Minimum number of samples required to split an internal node	40
	min_samples_leaf	Minimum number of samples required to be at a leaf node	80
	max_features	Number of features to consider when looking for the best split	$\sqrt{\text{n\_features}}$
	min_weight_fraction_leaf	minimum weighted fraction of the sum total of weights	0
Extreme Gradient Boosting‡	max_depth	Maximum tree depth for base learners	4
	learning_rate	Boosting learning rate	0.1
	n_estimators	Number of boosted trees to fit	120
	objective	learning task and the corresponding learning objective	binary:logistic
	booster	Which booster to use	gbtree
	gamma	Minimum loss reduction required to make a further partition on a leaf node of the tree	0.1
	min_child_weight	Minimum sum of instance weight(hessian) needed in a child	10
	max_delta_step	Maximum delta step we allow each tree's weight estimation to be	0
	subsample	Subsample ratio of the training instance	1.0
	colsample_bytree	Subsample ratio of columns when constructing each tree	0.8
	reg_alpha	L1 regularization term on weights	0.5
	reg_lambda	L2 regularization term on weights	2.5
	scale_pos_weight	Balancing of positive and negative weights	1
	base_score	Initial prediction score of all instances, global bias	0.5

	missing	Value in the data which needs to be present as a missing value	None
(Continued)			
Logistic Regression†	penalty	Norm used in the penalization	none
	C	Inverse of regularization strength	NA
	tol	Tolerance for stopping criteria	0.0001
Deep Neural Network§	-	Number of hidden layers	5
	-	Number of neurons in each hidden layer	12
	-	Activation function in the hidden layers	Leaky ReLU
	-	Activation function in the output layer	sigmoid
	-	Loss function	sigmoid cross entropy
	-	Optimizer	Adam optimizer
	-	Number of iterations	5000
	learning_rate	Learning rate	$0.01 \times 0.95^{\lfloor \frac{iteration}{500} \rfloor}$
	keep_prob	With probability keep_prob, outputs the input element scaled up by 1 / keep_prob, otherwise outputs 0	0.9 in model training and 1.0 in model testing

† Implemented in in Python 3.6 using scikit-learn (version 0.20.0)
‡ Implemented in Python 3.6 using xgboost (version 0.80)
§ Implemented in Python 3.6 using TensorFlow (version 1.10.0)
