# Supplementary material for: Predicting Breast Cancer in Chinese Women Using Machine Learning Techniques: Algorithm Development
Source: JMIR Med Inform. 2020 Jun 8;8(6):e17364. doi: 10.2196/17364 (PMC7308891; doi:10.2196/17364)
Supplement: Multimedia Appendix 2 [file medinform_v8i6e17364_app2.pdf]

In [4]:
```python
## import required packages ##
import numpy as np
from sklearn.metrics import roc_auc_score
import pandas as pd
from xgboost.sklearn import XGBClassifier
from sklearn.ensemble import RandomForestClassifier
from sklearn.linear_model import LogisticRegression
from sklearn.model_selection import KFold
import tensorflow as tf
from sklearn.preprocessing import OneHotEncoder
from tqdm import tqdm_notebook
import warnings
warnings.filterwarnings("ignore")

### define a function (ses_spe) to calculte sensitivity, specificity,
#and accuracy, and return these results ###
def ses_spe (real_value,pre_value,cut_value=0.5):
    data1=np.concatenate((real_value,pre_value),axis=1)
    real_pred=pd.DataFrame(data1,columns=['real','prediction'])
    real_pred['prediction2']=real_pred.prediction.apply(lambda x: 1 if x>cut_value else 0)
    TP_df=real_pred[real_pred.real==1][real_pred.prediction2==1]
    FP_df=real_pred[real_pred.real==0][real_pred.prediction2==1]
    TN_df=real_pred[real_pred.real==0][real_pred.prediction2==0]
    FN_df=real_pred[real_pred.real==1][real_pred.prediction2==0]
    TP=TP_df.count()[0]
    FP=FP_df.count()[0]
    TN=TN_df.count()[0]
    FN=FN_df.count()[0]
    ses=TP/(TP+FN)
    spe=TN/(TN+FP)
    ac=(TP+TN)/(TP+TN+FP+FN)
    return ses,spe,ac

## load the dataset ##
dataset=np.loadtxt("direction of the dataset",delimiter=',',skiprows=1)
## one-hot one-hot coding ##
enc = OneHotEncoder()
## position of the categorical independent variables that need to be transformed ##
enc.fit(dataset[:,[1,6,8,11]])
trans=enc.transform(dataset[:,[1,6,8,11]]).toarray()
## combine with the rest of the dataset ##
dataset_final=np.concatenate((dataset[:,[0,2,3,4,5,7,10]],trans),axis=1)

## four empty list to save the results for each ML model ##
result_dnn=[]
result_xgb=[]
result_rf=[]
result_lr=[]

## start loop 10 times 5-fold cross-validation ##
for i in tqdm_notebook(range(10),desc='repeat 10 times',leave=False):
    ## split the dataset into 5 subsets##
    ## random_state is defined so that the results are repeatable ##
    fold=KFold(n_splits=5,shuffle=True,random_state=i)
    for train_index,test_index in fold.split(dataset_final):
        ## train set ##
        train=dataset_final[train_index]
        ## test set ##
        test=dataset_final[test_index]
        ## independent variables in the train set ##
        x_train=train[:,1:15]
        ## independent variables in the test set ##
        x_test=test[:,1:15]
        ## dependent variables in the train set ##
        y_train=train[:,0][:,np.newaxis]
        ## dependent variables in the test set ##
        y_test=test[:,0][:,np.newaxis]
        ##DNN model##
        tf.reset_default_graph()
        ## random_seed is defined so that the results are repeatable ##
        tf.set_random_seed(i)
        ## length of the array ##
        n_variables = 14
        ## number of nodes ##
        n = 12
        x=tf.placeholder(tf.float32,[None,n_variables])
        y=tf.placeholder(tf.float32,[None,1])
        prob=tf.placeholder(tf.float32)
        ## learning rate ##
        lr=tf.Variable(0.01,dtype=tf.float32)
        ## define the structure of the newtwork ##
        w1=tf.Variable(tf.truncated_normal([n_variables,n],stddev=0.1))
        b1=tf.Variable(tf.zeros([1,n])+0.1)
        l1=tf.nn.leaky_relu(tf.matmul(x,w1)+b1)
        ## dropput is used ##
        l1_drop=tf.nn.dropout(l1,prob)

        w2=tf.Variable(tf.truncated_normal([n,n],stddev=0.1))
```

```
        b2=tf.Variable(tf.zeros([1,n])+0.1)
        l2=tf.nn.leaky_relu(tf.matmul(l1_drop,w2)+b2)
        ## dropput is used ##
        l2_drop=tf.nn.dropout(l2,prob)

        w3=tf.Variable(tf.truncated_normal([n,n],stddev=0.1))
        b3=tf.Variable(tf.zeros([1,n])+0.1)
        l3=tf.nn.leaky_relu(tf.matmul(l2_drop,w3)+b3)
        ## dropput is used ##
        l3_drop=tf.nn.dropout(l3,prob)

        w4=tf.Variable(tf.truncated_normal([n,n],stddev=0.1))
        b4=tf.Variable(tf.zeros([1,n])+0.1)
        l4=tf.nn.leaky_relu(tf.matmul(l3_drop,w4)+b4)
        ## dropput is used ##
        l4_drop=tf.nn.dropout(l4,prob)

        w5=tf.Variable(tf.truncated_normal([n,n],stddev=0.1))
        b5=tf.Variable(tf.zeros([1,n])+0.1)
        l5=tf.nn.leaky_relu(tf.matmul(l4_drop,w5)+b5)
        ## dropput is used ##
        l5_drop=tf.nn.dropout(l5,prob)

        w6=tf.Variable(tf.truncated_normal([n,1],stddev=0.1))
        b6=tf.Variable(tf.zeros([1,1])+0.1)
        prediction=tf.nn.sigmoid(tf.matmul(l5_drop,w6)+b6)

        ## define the loss function and optimizer ##
        loss=tf.reduce_mean(tf.nn.sigmoid_cross_entropy_with_logits(labels=y, logits=(tf.matmul(l5_drop,w6)+b6)
        train=tf.train.AdamOptimizer(lr).minimize(loss)
        init=tf.global_variables_initializer()

        ## start model fitting ##
        with tf.Session() as sess:
            sess.run(init)
            ## 5000 iterations ##
            for step in range(5001):
                sess.run(train,feed_dict={x:x_train,y:y_train,prob:0.9})
                ## when comes to last iteration calculate the auc and predicted probability##
                if step%5000 == 0:
                    predic_test=sess.run(prediction,feed_dict={x:x_test,prob:1.0})
                    predic_train=sess.run(prediction,feed_dict={x:x_train,prob:1.0})
                    auc_test = roc_auc_score(y_test,predic_test)
                    auc_train = roc_auc_score(y_train,predic_train)
                ## for every 500 iterations, reduce learning rate ##
                if step%500 == 0:
                    sess.run(tf.assign(lr,0.01*(0.95**(step//500))))
        ## calculate the performance on the train set ##
        ses_train,spe_train,ac_train=ses_spe(y_train,predic_train)
        ## calculate the performance on the test set ##
        ses_test,spe_test,ac_test=ses_spe(y_test,predic_test)
        ## save the results ##
        result_dnn.append([auc_train,ses_train,spe_train,ac_train,auc_test,ses_test,spe_test,ac_test])
        ## print results when finished ##
        print('ANN-'+str(i),'Train AUC:',auc_train,'Test AUC:',auc_test)
        ##XGBoost model##
        ## seed is defined so that the results are repeatable ##
        xgb1 = XGBClassifier(learning_rate =0.1,n_estimators=120,n_jobs=6,max_depth=4,
                             min_child_weight=10,seed=i,gamma=0.1,subsample=1.0,
                             colsample_bytree=0.8,
                             objective= 'binary:logistic',reg_alpha=0.5,reg_lambda=2.5)
        ## fit the model using train set ##
        xgb1.fit(x_train,y_train)
        ## calculate the predicted probability ##
        predic_train=xgb1.predict_proba(x_train)[:,1]
        predic_train=predic_train[:,np.newaxis]
        ## calculate the predicted probability ##
        predic_test=xgb1.predict_proba(x_test)[:,1]
        predic_test=predic_test[:,np.newaxis]
        ## calculate the auc ##
        auc_train=roc_auc_score(y_train,predic_train)
        auc_test=roc_auc_score(y_test,predic_test)
        ## calculate the performance on the train set ##
        ses_train,spe_train,ac_train=ses_spe(y_train,predic_train)
        ## calculate the performance on the test set ##
        ses_test,spe_test,ac_test=ses_spe(y_test,predic_test)
        ## save the results ##
        result_xgb.append([auc_train,ses_train,spe_train,ac_train,auc_test,ses_test,spe_test,ac_test])
        ## print results when finished ##
        print('XGBoost-'+str(i),'Train AUC:',auc_train,'Test AUC:',auc_test)
        ##Random Forest model##
        ## random_state is defined so that the results are repeatable ##
        rf=RandomForestClassifier(n_estimators=320,criterion='gini',random_state=i,
                                  min_samples_leaf=80,min_samples_split=40,n_jobs=6)
        ## fit the model using train set ##
        rf.fit(x_train,y_train)
        ## calculate the predicted probability ##
        predic_test=rf.predict_proba(x_test)[:,1]
```

```
        predic_train=rf.predict_proba(x_train)[:,1]
        predic_test=predic_test[:,np.newaxis]
        predic_train=predic_train[:,np.newaxis]
        ## calculate the auc ##
        auc_test=roc_auc_score(y_test,predic_test)
        auc_train=roc_auc_score(y_train,predic_train)
        ## calculate the performance on the train set ##
        ses_train,spe_train,ac_train=ses_spe(y_train,predic_train)
        ## calculate the performance on the test set ##
        ses_test,spe_test,ac_test=ses_spe(y_test,predic_test)
        ## save the results ##
        result_rf.append([auc_train,ses_train,spe_train,ac_train,auc_test,ses_test,spe_test,ac_test])
        ## print results when finished ##
        print('RF-'+str(i),'Train AUC:',auc_train,'Test AUC:',auc_test)
        ##Logistice regression model##
        ## random_state is defined so that the results are repeatable ##
        lr=LogisticRegression(random_state=i,n_jobs=6,penalty='none')
        ## fit the model using train set ##
        lr.fit(x_train,y_train)
        ## calculate the predicted probability ##
        predic_test=lr.predict_proba(x_test)[:,1]
        predic_train=lr.predict_proba(x_train)[:,1]
        predic_test=predic_test[:,np.newaxis]
        predic_train=predic_train[:,np.newaxis]
        ## calculate the auc ##
        auc_test=roc_auc_score(y_test,predic_test)
        auc_train=roc_auc_score(y_train,predic_train)
        ## calculate the performance on the train set ##
        ses_train,spe_train,ac_train=ses_spe(y_train,predic_train)
        ## calculate the performance on the test set ##
        ses_test,spe_test,ac_test=ses_spe(y_test,predic_test)
        ## save the results ##
        result_lr.append([auc_train,ses_train,spe_train,ac_train,auc_test,ses_test,spe_test,ac_test])
        ## print results when finished ##
        print('LR-'+str(i),'Train AUC:',auc_train,'Test AUC:',auc_test)
```

A Jupyter widget could not be displayed because the widget state could not be found. This could happen if the kernel storing the widget is no longer available, or if the widget state was not saved in the notebook. You may be able to create the widget by running the appropriate cells.

```
ANN-0 Train AUC: 0.7449184999138322 Test AUC: 0.7305673132495837
XGBoost-0 Train AUC: 0.7715926607069278 Test AUC: 0.7438529331549248
RF-0 Train AUC: 0.7430629853013933 Test AUC: 0.7298707129544025
LR-0 Train AUC: 0.6340363816960541 Test AUC: 0.6293938740526974
ANN-0 Train AUC: 0.7440035509811527 Test AUC: 0.7270703536382516
XGBoost-0 Train AUC: 0.7706127761983608 Test AUC: 0.7412348303658428
RF-0 Train AUC: 0.7426603355725179 Test AUC: 0.7291217606125924
LR-0 Train AUC: 0.6353152169472539 Test AUC: 0.6255324750740642
ANN-0 Train AUC: 0.7440141684757076 Test AUC: 0.7278577629224564
XGBoost-0 Train AUC: 0.7690935388384125 Test AUC: 0.7463807378186693
RF-0 Train AUC: 0.7423999824530619 Test AUC: 0.7307678869158345
LR-0 Train AUC: 0.6294315554979134 Test AUC: 0.6388508319701739
ANN-0 Train AUC: 0.740058836449682 Test AUC: 0.7308150361612749
XGBoost-0 Train AUC: 0.771112229158835 Test AUC: 0.74347960244251
RF-0 Train AUC: 0.7420097203499794 Test AUC: 0.7273898527958551
LR-0 Train AUC: 0.6335223179210916 Test AUC: 0.6279409333034123
ANN-0 Train AUC: 0.7424067257950557 Test AUC: 0.7293199670506813
XGBoost-0 Train AUC: 0.7728213304130545 Test AUC: 0.7400287419745154
RF-0 Train AUC: 0.7437516615037978 Test AUC: 0.7252335716509737
LR-0 Train AUC: 0.6307192986163278 Test AUC: 0.6336477181808507
ANN-1 Train AUC: 0.73275792163817 Test AUC: 0.7398154572968184
XGBoost-1 Train AUC: 0.7679542919574929 Test AUC: 0.7579879924214463
RF-1 Train AUC: 0.7384058259022905 Test AUC: 0.7438502989591791
LR-1 Train AUC: 0.6271646731228149 Test AUC: 0.6370881622007333
ANN-1 Train AUC: 0.7396603400137569 Test AUC: 0.7155971746196113
XGBoost-1 Train AUC: 0.7724523885991045 Test AUC: 0.7264822321135034
RF-1 Train AUC: 0.7459876697023281 Test AUC: 0.7180021044674162
LR-1 Train AUC: 0.6334425408497637 Test AUC: 0.6297124124204919
ANN-1 Train AUC: 0.7406628803959281 Test AUC: 0.7200957444085935
XGBoost-1 Train AUC: 0.7742934119873555 Test AUC: 0.7362439390747825
RF-1 Train AUC: 0.7453851979824604 Test AUC: 0.7223392464471118
LR-1 Train AUC: 0.6359365175168856 Test AUC: 0.6269248297625634
ANN-1 Train AUC: 0.7307542859653101 Test AUC: 0.7276199659012159
XGBoost-1 Train AUC: 0.7687723207171817 Test AUC: 0.7593594468594469
RF-1 Train AUC: 0.7383904276508827 Test AUC: 0.7452971437346437
LR-1 Train AUC: 0.6356103505248564 Test AUC: 0.6254343559031059
ANN-1 Train AUC: 0.7449332574737554 Test AUC: 0.7142972876472834
XGBoost-1 Train AUC: 0.773425950668391 Test AUC: 0.7283293729454206
RF-1 Train AUC: 0.7461362487721723 Test AUC: 0.7136942416370151
LR-1 Train AUC: 0.6313151468422928 Test AUC: 0.6335967299097525
ANN-2 Train AUC: 0.7397802972478678 Test AUC: 0.7343877340105119
XGBoost-2 Train AUC: 0.7716151364511755 Test AUC: 0.7497632827417862
RF-2 Train AUC: 0.7418485894674048 Test AUC: 0.7338749286403276
LR-2 Train AUC: 0.6344074779927482 Test AUC: 0.6237822102797299
ANN-2 Train AUC: 0.7394387379987757 Test AUC: 0.7392815031928379
XGBoost-2 Train AUC: 0.7700353307431299 Test AUC: 0.7460195330031574
RF-2 Train AUC: 0.7412571407477135 Test AUC: 0.7379382770485736
LR-2 Train AUC: 0.6334869613099907 Test AUC: 0.6297663736289694
ANN-2 Train AUC: 0.7459119296603827 Test AUC: 0.7126223022609856
```

```
XGBoost-2 Train AUC: 0.7725901663954883 Test AUC: 0.7258260725724903
RF-2 Train AUC: 0.7464068646406588 Test AUC: 0.7092140359721509
LR-2 Train AUC: 0.6283772156096559 Test AUC: 0.6455646753359281
ANN-2 Train AUC: 0.744718919699130l Test AUC: 0.7345342228154728
XGBoost-2 Train AUC: 0.7687201777745184 Test AUC: 0.7495909917784918
RF-2 Train AUC: 0.7416678961531613 Test AUC: 0.7332880028192529
LR-2 Train AUC: 0.6320785044456549 Test AUC: 0.6224546497983998
ANN-2 Train AUC: 0.7396059872289084 Test AUC: 0.7303556786703601
XGBoost-2 Train AUC: 0.7715501560554419 Test AUC: 0.7350155740227763
RF-2 Train AUC: 0.7423820473927749 Test AUC: 0.7279157894736842
LR-2 Train AUC: 0.6353683920956187 Test AUC: 0.6317188057863958
ANN-3 Train AUC: 0.7423414783788596 Test AUC: 0.7281133843633845
XGBoost-3 Train AUC: 0.7682588896180356 Test AUC: 0.7450825497700498
RF-3 Train AUC: 0.7422385152938481 Test AUC: 0.7308164808164809
LR-3 Train AUC: 0.6329952204455954 Test AUC: 0.6201657295407296
ANN-3 Train AUC: 0.7444531404343266 Test AUC: 0.7249737895187608
XGBoost-3 Train AUC: 0.76835059634816 Test AUC: 0.7465918991154885
RF-3 Train AUC: 0.742327254439564 Test AUC: 0.7325416045244457
LR-3 Train AUC: 0.6339401542119864 Test AUC: 0.6281495646352928
ANN-3 Train AUC: 0.7404992726202191 Test AUC: 0.7268633755864802
XGBoost-3 Train AUC: 0.7689659394304442 Test AUC: 0.7412961765478796
RF-3 Train AUC: 0.7415032535979074 Test AUC: 0.7282078386329388
LR-3 Train AUC: 0.6307059321520321 Test AUC: 0.6400042715736616
ANN-3 Train AUC: 0.745659609852018 Test AUC: 0.7178736535163626
XGBoost-3 Train AUC: 0.7724928199284822 Test AUC: 0.7317920759524077
RF-3 Train AUC: 0.7459187415248084 Test AUC: 0.7170491753249182
LR-3 Train AUC: 0.636800266037954 Test AUC: 0.6238737874017763
ANN-3 Train AUC: 0.7406242552842294 Test AUC: 0.7404351734413674
XGBoost-3 Train AUC: 0.7715431931763644 Test AUC: 0.7471565959437994
RF-3 Train AUC: 0.7408285311661358 Test AUC: 0.7325963870999521
LR-3 Train AUC: 0.6290118226877348 Test AUC: 0.6413131549458804
ANN-4 Train AUC: 0.7415972792396981 Test AUC: 0.7257145289605804
XGBoost-4 Train AUC: 0.773421675086881 Test AUC: 0.7363415447939459
RF-4 Train AUC: 0.7440859055670626 Test AUC: 0.7218989014969207
LR-4 Train AUC: 0.634932810042363 Test AUC: 0.618060538237966
ANN-4 Train AUC: 0.7413964916577037 Test AUC: 0.7227225865715743
XGBoost-4 Train AUC: 0.7728771692554572 Test AUC: 0.7356962489429268
RF-4 Train AUC: 0.7442825920556827 Test AUC: 0.7191627882680166
LR-4 Train AUC: 0.6353034404126969 Test AUC: 0.6175770800931211
ANN-4 Train AUC: 0.7447441968779303 Test AUC: 0.7255917538398533
XGBoost-4 Train AUC: 0.7715489666111293 Test AUC: 0.7357660608351694
RF-4 Train AUC: 0.7432171550181748 Test AUC: 0.7250269247504908
LR-4 Train AUC: 0.634688866643861 Test AUC: 0.6318456301179176
ANN-4 Train AUC: 0.7433754424114438 Test AUC: 0.735854478987769
XGBoost-4 Train AUC: 0.7680959355986767 Test AUC: 0.7476114398499873
RF-4 Train AUC: 0.7415138070520015 Test AUC: 0.7348737669005849
LR-4 Train AUC: 0.6294939988333629 Test AUC: 0.6397634263157168
ANN-4 Train AUC: 0.7404411085105835 Test AUC: 0.7309848132636215
XGBoost-4 Train AUC: 0.769198497753258 Test AUC: 0.7481553404752429
RF-4 Train AUC: 0.741094931247498 Test AUC: 0.7338026531971144
LR-4 Train AUC: 0.6288780038898724 Test AUC: 0.6442998518559377
ANN-5 Train AUC: 0.7417244917440107 Test AUC: 0.717614452328714
XGBoost-5 Train AUC: 0.7722453841095992 Test AUC: 0.7308865521528191
RF-5 Train AUC: 0.744555532839043 Test AUC: 0.7187001388851977
LR-5 Train AUC: 0.6351828621489082 Test AUC: 0.6222177436421388
ANN-5 Train AUC: 0.7407304630684685 Test AUC: 0.7356745068389852
XGBoost-5 Train AUC: 0.7704157519817489 Test AUC: 0.7521281381854982
RF-5 Train AUC: 0.7415819198785234 Test AUC: 0.7326428882233995
LR-5 Train AUC: 0.630695882441249 Test AUC: 0.631749153863874
ANN-5 Train AUC: 0.7423937190056 Test AUC: 0.7357819520381097
XGBoost-5 Train AUC: 0.7701705123825017 Test AUC: 0.7392732882880666
RF-5 Train AUC: 0.7425752179171295 Test AUC: 0.7316415111981613
LR-5 Train AUC: 0.630932961008312 Test AUC: 0.6417188722607441
ANN-5 Train AUC: 0.7434108449866605 Test AUC: 0.7282214906494764
XGBoost-5 Train AUC: 0.7709689596536038 Test AUC: 0.7448295483632152
RF-5 Train AUC: 0.7426109919352982 Test AUC: 0.7337520132470673
LR-5 Train AUC: 0.6321235081027863 Test AUC: 0.6344455251769098
ANN-5 Train AUC: 0.7431890432554664 Test AUC: 0.720430830358761
XGBoost-5 Train AUC: 0.7723904423508299 Test AUC: 0.7420353855912505
RF-5 Train AUC: 0.7432716570679115 Test AUC: 0.7261076342103396
LR-5 Train AUC: 0.6339810418216894 Test AUC: 0.6263671213231528
ANN-6 Train AUC: 0.7458264325293793 Test AUC: 0.7151582407740043
XGBoost-6 Train AUC: 0.7755861784210591 Test AUC: 0.7276870912338893
RF-6 Train AUC: 0.7475890330605497 Test AUC: 0.7124469126932181
LR-6 Train AUC: 0.6324128692941433 Test AUC: 0.6378567245562319
ANN-6 Train AUC: 0.7385925428419913 Test AUC: 0.7354812659112957
XGBoost-6 Train AUC: 0.7714701714233398 Test AUC: 0.751673240463071
RF-6 Train AUC: 0.740717837735137 Test AUC: 0.7389727642936985
LR-6 Train AUC: 0.6363927444180649 Test AUC: 0.6194653312257792
ANN-6 Train AUC: 0.7375622033648344 Test AUC: 0.741678110679602
XGBoost-6 Train AUC: 0.7675892759525564 Test AUC: 0.7539089302678145
RF-6 Train AUC: 0.7399837516483534 Test AUC: 0.7442634731717882
LR-6 Train AUC: 0.6315626584097387 Test AUC: 0.6346834807519086
ANN-6 Train AUC: 0.7413625914148416 Test AUC: 0.719936928450997
XGBoost-6 Train AUC: 0.7732560156987581 Test AUC: 0.7371602940583017
RF-6 Train AUC: 0.7459880062866401 Test AUC: 0.7180786406152035
LR-6 Train AUC: 0.6312060806539386 Test AUC: 0.6337366841570136
ANN-6 Train AUC: 0.7415807228008413 Test AUC: 0.7325158791825459
```

```
XGBoost-6 Train AUC: 0.7727682597211547 Test AUC: 0.7385000185000186
RF-6 Train AUC: 0.7437309600075105 Test AUC: 0.7282645749312415
LR-6 Train AUC: 0.6316569609025184 Test AUC: 0.6251003317669984
ANN-7 Train AUC: 0.743401176395962 Test AUC: 0.7274400476370919
XGBoost-7 Train AUC: 0.7708924166124785 Test AUC: 0.7415776341884717
RF-7 Train AUC: 0.7439238163548485 Test AUC: 0.7266553643400935
LR-7 Train AUC: 0.6313602217510633 Test AUC: 0.6307308455091706
ANN-7 Train AUC: 0.7431325884604487 Test AUC: 0.722928294327425
XGBoost-7 Train AUC: 0.7706109407652857 Test AUC: 0.7379934092276723
RF-7 Train AUC: 0.7433608863903239 Test AUC: 0.7224400433738214
LR-7 Train AUC: 0.6361348769772259 Test AUC: 0.6236752222612889
ANN-7 Train AUC: 0.7443410209464962 Test AUC: 0.7257040000787418
XGBoost-7 Train AUC: 0.7739417421693511 Test AUC: 0.7289469280891355
RF-7 Train AUC: 0.7438982856990977 Test AUC: 0.7227147680072442
LR-7 Train AUC: 0.6331246316948479 Test AUC: 0.627593308923404
ANN-7 Train AUC: 0.7402921145781272 Test AUC: 0.7417832882609959
XGBoost-7 Train AUC: 0.7708321476227653 Test AUC: 0.7549380070637959
RF-7 Train AUC: 0.7419342096301007 Test AUC: 0.737515455132089
LR-7 Train AUC: 0.6329201133286009 Test AUC: 0.6316013733516578
ANN-7 Train AUC: 0.745244326118379 Test AUC: 0.7360596488947195
XGBoost-7 Train AUC: 0.7731304244990319 Test AUC: 0.7377170277393656
RF-7 Train AUC: 0.743883569530088 Test AUC: 0.7318599380680542
LR-7 Train AUC: 0.6296195697724541 Test AUC: 0.6428007560267426
ANN-8 Train AUC: 0.736906141531295 Test AUC: 0.7409594885877108
XGBoost-8 Train AUC: 0.7677503375265543 Test AUC: 0.7560198718516551
RF-8 Train AUC: 0.740965504367788 Test AUC: 0.7408531904213542
LR-8 Train AUC: 0.6325316688011766 Test AUC: 0.6313180972628222
ANN-8 Train AUC: 0.7461050732718431 Test AUC: 0.7102980445654432
XGBoost-8 Train AUC: 0.773041569777368 Test AUC: 0.7310226783951401
RF-8 Train AUC: 0.7460953215350647 Test AUC: 0.7097510406116602
LR-8 Train AUC: 0.6374600817115559 Test AUC: 0.6183177180067895
ANN-8 Train AUC: 0.740688385972751 Test AUC: 0.7444977725270128
XGBoost-8 Train AUC: 0.7689427179830054 Test AUC: 0.7502057643555097
RF-8 Train AUC: 0.7400739858136689 Test AUC: 0.744507371581875
LR-8 Train AUC: 0.6295644329863608 Test AUC: 0.6401973959487066
ANN-8 Train AUC: 0.742660879815843 Test AUC: 0.7237005026629226
XGBoost-8 Train AUC: 0.7741761089831953 Test AUC: 0.7392572655806313
RF-8 Train AUC: 0.7437347398393517 Test AUC: 0.7280831780659043
LR-8 Train AUC: 0.6306137010978856 Test AUC: 0.6353865543819864
ANN-8 Train AUC: 0.745080798757571 Test AUC: 0.722524755401268
XGBoost-8 Train AUC: 0.7754745006396475 Test AUC: 0.733065995358239
RF-8 Train AUC: 0.7444805055098443 Test AUC: 0.7216113273938758
LR-8 Train AUC: 0.6328292986165327 Test AUC: 0.6296107304271761
ANN-9 Train AUC: 0.7386601403320477 Test AUC: 0.7390334486463278
XGBoost-9 Train AUC: 0.769307177501687 Test AUC: 0.7488357917354754
RF-9 Train AUC: 0.7404762178703617 Test AUC: 0.7389460530164048
LR-9 Train AUC: 0.6315593874444156 Test AUC: 0.63403412319288
ANN-9 Train AUC: 0.7418151505281384 Test AUC: 0.7339019961022855
XGBoost-9 Train AUC: 0.7690022232741662 Test AUC: 0.7532207819051555
RF-9 Train AUC: 0.7417447349513392 Test AUC: 0.7304523711096675
LR-9 Train AUC: 0.6288121006015563 Test AUC: 0.6442550542333509
ANN-9 Train AUC: 0.7401534286261507 Test AUC: 0.7321000959622056
XGBoost-9 Train AUC: 0.7676988862310765 Test AUC: 0.7506151423439384
RF-9 Train AUC: 0.7416393346906163 Test AUC: 0.7354334292955391
LR-9 Train AUC: 0.6328796242741048 Test AUC: 0.6241140719962599
ANN-9 Train AUC: 0.7460108962655219 Test AUC: 0.7264289701491213
XGBoost-9 Train AUC: 0.7713769994047353 Test AUC: 0.7401304357671039
RF-9 Train AUC: 0.7430722731264929 Test AUC: 0.7253571010160974
LR-9 Train AUC: 0.6338369344024511 Test AUC: 0.620462338628933
ANN-9 Train AUC: 0.7495290495395871 Test AUC: 0.7043034797445062
XGBoost-9 Train AUC: 0.776232656524765 Test AUC: 0.7174783673453304
RF-9 Train AUC: 0.7482315001674897 Test AUC: 0.7099446180398213
LR-9 Train AUC: 0.6366357335866764 Test AUC: 0.6299949169172089
```

In [ ]:
