# Supplementary material for: Predicting Breast Cancer in Chinese Women Using Machine Learning Techniques: Algorithm Development
Source: JMIR Med Inform. 2020 Jun 8;8(6):e17364. doi: 10.2196/17364 (PMC7308891; doi:10.2196/17364)
Supplement: Multimedia Appendix 3 [file medinform_v8i6e17364_app3.pdf]

- Hyper-parameters tuning for each ML algorithm using grid search and 10-fold cross-validation (final configuration of hyper-parameters for each ML algorithm is shown in Supplemental File 1)
- For K = 1 to 10:
  - Randomly divide the dataset into 5 folds with approximately equal sample size
  - For J = 1 to 5 (5-fold cross-validation):
    - Choose the J_{th} fold as validation set and the remaining four folds as training set
    - Develop four ML models (XGBoost, RF, DNN and LR) using the training set
    - Calculate AUC (AUC_0) for each ML model using the validation set
    - For I = 1 to 10:
      - Permute the values of the I_{th} variable in the testing set and calculate the corresponding AUC (AUC_i) for each ML model
      - Calculate AUC decrease for the I_{th} variable for each ML model: $1 - \frac{AUC_i}{AUC_0}$
- Calculate the mean decrease in AUC for each variable and each ML algorithm
- Ranking each variable in the four ML algorithms according to their mean decrease in AUC
