# Supplementary material for: Predicting Breast Cancer in Chinese Women Using Machine Learning Techniques: Algorithm Development
Source: JMIR Med Inform. 2020 Jun 8;8(6):e17364. doi: 10.2196/17364 (PMC7308891; doi:10.2196/17364)
Supplement: Multimedia Appendix 4 [file medinform_v8i6e17364_app4.pdf]

Comparison	AUC		Sensitivity		Specificity		Accuracy	
	**Difference**	**P-value‡**	**Difference**	**P-value‡**	**Difference**	**P-value‡**	**Difference**	**P-value‡**
DNN vs. LR	0.097	<0.001†	0.146	<0.001†	0.018	0.35	0.083	<0.001†
XGBoost vs. LR	0.111	<0.001†	0.16	<0.001†	0.025	0.02	0.093	<0.001†
RF vs. LR	0.097	<0.001†	0.154	<0.001†	0.016	0.17	0.085	<0.001†
XGBoost vs. DNN	0.014	<0.001†	0.014	0.45	0.007	0.71	0.01	0.002†
DNN vs. RF	0	0.98	-0.008	0.64	0.002	0.90	-0.002	0.34
XGBoost vs. RF	0.014	<0.001†	0.006	0.28	0.009	0.15	0.008	0.01

† A P-value < 0.008 is considered to be statistically significant

‡ P-values were derived from the pair-wise corrected resampled t-test
